# TetR-family transcription factors in Gram-negative bacteria: conservation, variation and implications for efflux-mediated antimicrobial resistance

**DOI:** 10.1186/s12864-019-6075-5

**Published:** 2019-10-12

**Authors:** A. L. Colclough, J. Scadden, J. M. A. Blair

**Affiliations:** Institute of Microbiology and Infection, Biosciences Building, University of Birmingham, Edgbaston, Birmingham, B15 2TT UK

**Keywords:** TetR-family, Regulation, Antimicrobial resistance, Conservation

## Abstract

**Background:**

TetR-family transcriptional regulators (TFTRs) are DNA binding factors that regulate gene expression in bacteria. Well-studied TFTRs, such as AcrR, which regulates efflux pump expression, are usually encoded alongside target operons. Recently, it has emerged that there are many TFTRs which act as global multi-target regulators. Our classical view of TFTRs as simple, single-target regulators therefore needs to be reconsidered. As some TFTRs regulate essential processes (e.g. metabolism) or processes which are important determinants of resistance and virulence (e.g. biofilm formation and efflux gene expression) and as TFTRs are present throughout pathogenic bacteria, they may be good drug discovery targets for tackling antimicrobial resistant infections. However, the prevalence and conservation of individual TFTR genes in Gram-negative species, has to our knowledge, not yet been studied.

**Results:**

Here, a wide-scale search for TFTRs in available proteomes of clinically relevant pathogens *Salmonella* and *Escherichia* species was performed and these regulators further characterised. The majority of identified TFTRs are involved in efflux regulation in both *Escherichia* and *Salmonella*. The percentage variance in TFTR genes of these genera was found to be higher in those regulating genes involved in efflux, bleach survival or biofilm formation than those regulating more constrained processes. Some TFTRs were found to be present in all strains and species of these two genera, whereas others (i.e. TetR) are only present in some strains and some (i.e. RamR) are genera-specific. Two further pathogens on the WHO priority pathogen list (*K. pneumoniae* and *P. aeruginosa*) were then searched for the presence of the TFTRs conserved in *Escherichia* and *Salmonella*.

**Conclusions:**

Through bioinformatics and literature analyses, we present that TFTRs are a varied and heterogeneous family of proteins required for the regulation of numerous important processes, with consequences to antimicrobial resistance and virulence, and that the roles and responses of these proteins are frequently underestimated.

**Electronic supplementary material:**

The online version of this article (10.1186/s12864-019-6075-5) contains supplementary material, which is available to authorized users.

## Background

The TetR-family of transcriptional regulators (TFTRs) are a large family of one-component signal transduction proteins, with over 200,000 sequences available on public databases. TFTRs are implicated in the regulation of many processes, including efflux regulation, cell division and the stress response [1, 2]. Some of these processes are essential for cell growth and survival and therefore these TFTRs could be targets for inhibiting bacterial growth. Other processes, such as efflux, are important for antimicrobial resistance and the negative regulation of these efflux systems is commonly regulated by TFTRs.

**Fig. 1:**
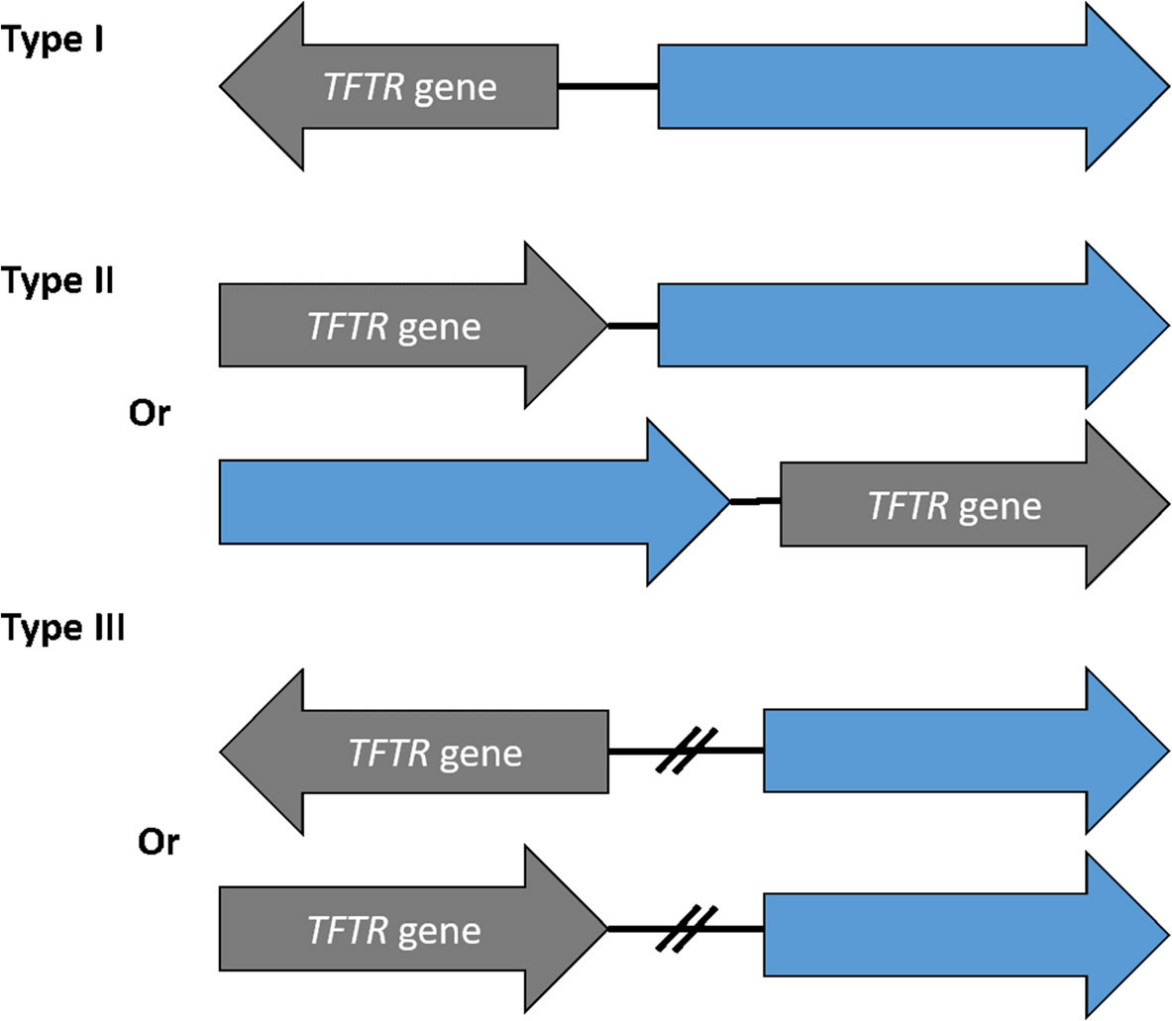
TFTR regulation classification proposed by Ahn et al. Current classification system of TFTRs as proposed by Ahn et al. Type I classification involves the TFTR gene regulating a divergently expressed target gene (i.e. AcrR). Type II TFTRs regulate genes directly up/downstream in the same orientation (i.e. ComR). Type III TFTRs regulate genes either up/downstream of the *TFTR* gene in any orientation and any location on the genome

TFTRs have a highly conserved helix-turn-helix (HTH) motif at the N-terminus and a variable ligand-binding C-terminal domain [3]. Many TFTRs act as repressors by binding palindromic sequences which overlap promoters, preventing the recruitment and binding of RNA polymerase and preventing transcription. Upon ligand binding, a conformational change occurs which releases the TFTR from target DNA, enabling transcription of target genes [2]. Some authors choose to classify TFTRs based on their location in relation to their target gene (Fig. 1) and it is believed that the majority of TFTRs regulate genes within 200 base pairs (bp) of the TFTR-encoding gene [4, 5]. A TFTR classification system proposed by Ahn et al.*,* describes three types of TFTR which bind targets which are either divergently encoded (Type I) encoded alongside (Type II) or neither I or II (Type III) [4]. Type I TFTRs are the most common (i.e. AcrR regulating *acrAB*) than type II TFTRs (i.e. ComR regulating *comAB*). Both Type I and II TFTRs are thought to act on local genes, whereas Type III TFTRs act globally and in any orientation (i.e. RutR).

There are numerous examples of TFTRs regulating local genes, such as AcrR regulating the adjacent *acrAB* efflux genes. However, some TFTRs are global regulators able to alter transcription of multiple targets throughout the genome, such as MtrR of *Neisseria gonorrhoea* [6]. In *Mycobacteria* the number of TFTRs has been shown to increase with genome size and while the number of TFTRs can vary between species, the majority of TFTRs in *Mycobacteria* are believed to regulate targets within 300 bp of the *tftr* gene [5]. However, it is now known that some TFTRs act to regulate multiple targets and can therefore act locally and globally and meaning they would fit into multiple categories of the classification system in Fig. 1. For example, the TFTR EnvR regulates the divergently encoded local efflux operon *acrEF*, but also binds upstream and regulates expression of the efflux operon *acrAB*, which is encoded separately on the genome. Some TFTRs with multiple targets may therefore not fit an individual classification of TFTR. Other TFTRs are activators [7] and some can act as both activators and repressors [8]. TFTRs have been identified which can bind multiple targets [9, 10] and intergenic regions [11]. Thus, although some TFTRs are known to be local repressors, the current classification system is, in some cases, oversimplifying these proteins.

Efflux genes are frequently encoded in operons and are often negatively regulated by TFTRs. The extrusion of antimicrobials by efflux pumps such as AcrAB is a key mechanism of antimicrobial resistance [12, 13]. Specifically, mutations resulting in non-functional efflux regulators can cause increased expression of efflux genes, for example mutations in AcrR [14, 15] and EnvR [16], increase the efflux of antimicrobials by the AcrAB efflux system. These regulators may also have additional roles, for example there is evidence that AcrR can bind upstream of, and influence expression of *flhC* and *flhD*, the master regulators of flagella expression [17].

While individual TFTRs have been studied in various Gram-negative bacteria and homologs of certain members of the TFTR family are known to be present in different species, it is not understood how conserved the TFTR family of proteins are across the Gram-negative bacteria, both in terms of which regulators and present/absent or their level of sequence conservation.

Here, phylogenomic analyses of the conservation of the TFTRs across two genera, *Escherichia* and *Salmonella* were compared on three levels: genera, species and strain, to evaluate the conservation of TFTRs. From these analyses, we identify which TFTR genes are core (i.e. present in all) for *Escherichia* and *Salmonella* genera and of these core genes, which are also present in *P. aeruginosa* and *K. pneumoniae*. For this analysis, the TetR HTH was used to search for the presence of TFTR genes and then these genes were grouped based on function, through searching literature for experimental evidence of biological roles.

## Results

### Patterns of TFTR presence and absence across *Escherichia* and *Salmonella* genera

Maximum-likelihood trees constructed using the sequence of *acrB* were overlaid with data on the presence/absence of accessory TFTRs in the *Escherichia* and *Salmonella* genera using Phandango [18]. This data was combined with predicted function of these TFTRs, which was ascertained through searching known targets in the literature and compiled in Table 1, below:

**Table 1:** Proposed biological roles of TFTRs of *Salmonella* and *Escherichia*. TFTRs present in all Gram-negative species tested are denoted as core**, while those not present in all species but present in all *Escherichia* and *Salmonella* are denoted as core*. The carriage of the remaining TFTRs found in *Salmonella* and *Escherichia* are listed (%, italicised for *Salmonella*). This data is combined with biological role as documented in literature. Known targets and ligands are included and targets known to be activated, not repressed, by the TFTR are in bold. A biological role was assigned from the literature if experimental evidence was provided (e.g. binding assays to show TFTR binding to promoter)

TFTR	Core/Accessory (%)	Pathway	Gene(s) or process regulated (organism)	Ligands	References
AcrR	Core**	Multidrug efflux (RND) Multidrug efflux (ABC) Multidrug efflux (MFS) Motility	*acrAB* (Enterobacteriales) ***flhDC***	Rhodamine 6 g Proflavin Ethidium bromide Ciprofloxacin	[19] [20] [21]
EnvR	Core**	Multidrug efflux (RND) Multidrug efflux (RND)	*acrAB* (Enterobacteriales) *acrEF* (Enterobacteriales)	No data available	[9]
NemR	Core**	Bleach survival	*nemAB*	Choline	[22]
SlmA	Core*	Cell division Chitin catabolism	FtsZ ring formation(Enterobacteriales) ***chb operon*** (*V. cholera*)	Target DNA sequences FtsZ protein	[23] [24] [25]
YbiH	Core*	Multidrug efflux (ABC) Membrane permeability	*ybhGFSR* (*E. coli*) ***rhlE***(*E. coli*)	Chloramphenicol Cephalosporin	[26]
BetI	Accessory (67%)	Glycine betaine synthesis	*betT* (Enterobacteriales) *betIBA* (Enterobacteriales)	Choline	[27]
EefR	Accessory (47%)	Multidrug efflux (RND)	*eefABC* (*Enterobacter spp.*, *K. pneumoniae*)	No data available	[28] [29]
FabR	Core *Accessory (93%)*	Fatty acid biosynthesis	*fabAB* (Enterobacteriales)	Unsaturated thioester	[30]
RamR	*Core*	Efflux regulation	*ramA* (Enterobacteriales)	Bile Berberine Ethidium bromide Dequalinium Crystal violet Rhodamine 6 g	[31] [32] [33]
RutR	Core *Accessory (93%)*	Pyrimidine utilisation Purine degradation Glutamine supply PH homeostasis	*rutABCDEFG* (*E. coli*) ***carAB*** (*E. coli*) *gadAXW* (*E. coli*) *gadIBC* (*E. coli*) *gly-hyi-glxR-ybbVW-allB-ybbY-glxK* (*E. coli*)	Uracil Thymine	[34] [11] [35]
TetR	Accessory (40%) *Accessory (20%)*	Multidrug efflux (ABC)	*tetAB* (Enterobacteriales)	Tetracycline	[36]
UidR	Accessory (67%)	Catalysis of beta-glucuronidase	*uidA* (*E. coli*)	No data available	[37]
U1	*Core*	No data available	No data available	No data available	
YbjK/ RcdA	Accessory (93%) *Accessory (80%)*	Biofilm formation Stress response	*csgD* (*E. coli*) *appY*, *sxy*, *ycgF*, *fimB* (*E. coli*)	No data available	[38]
YcfQ/ comR	Accessory (80%) *Core*	Copper transport	*comC* (*E. coli*)	Copper	[39]
YftA	Accessory (80%)	No data available	No data available	No data available	
YjdC	Accessory (67%) *Core*	Copper tolerance	*cadABC* (*E. coli)*	No data available	[40]
YjgJ/ bdcR	Accessory (60%) *Accessory (93%)*	Biofilm dispersal	*bdcA* (*E. coli*)	No data available	[41]

The TFTRs identified here are included based on the presence of the TetR HTH motif. SlmA contains this HTH and is therefore referred to by some authors as a TFTR. SlmA directly activates the transcription of the *chb* operon in *V. cholerae* [25], but is not believed to have any direct regulatory roles in *E. coli* [42]. In *E. coli*, SlmA acts as a nucleoid occlusion protein, interacting with target DNA and protein (FtsZ). Thus, although we include SlmA here, this is based on the presence of the HTH motif and not the assumption of direct regulatory roles in either *Salmonella* or *Escherichia.*

### TFTRs of *E. coli* and *Escherichia* species

**Fig. 2:**
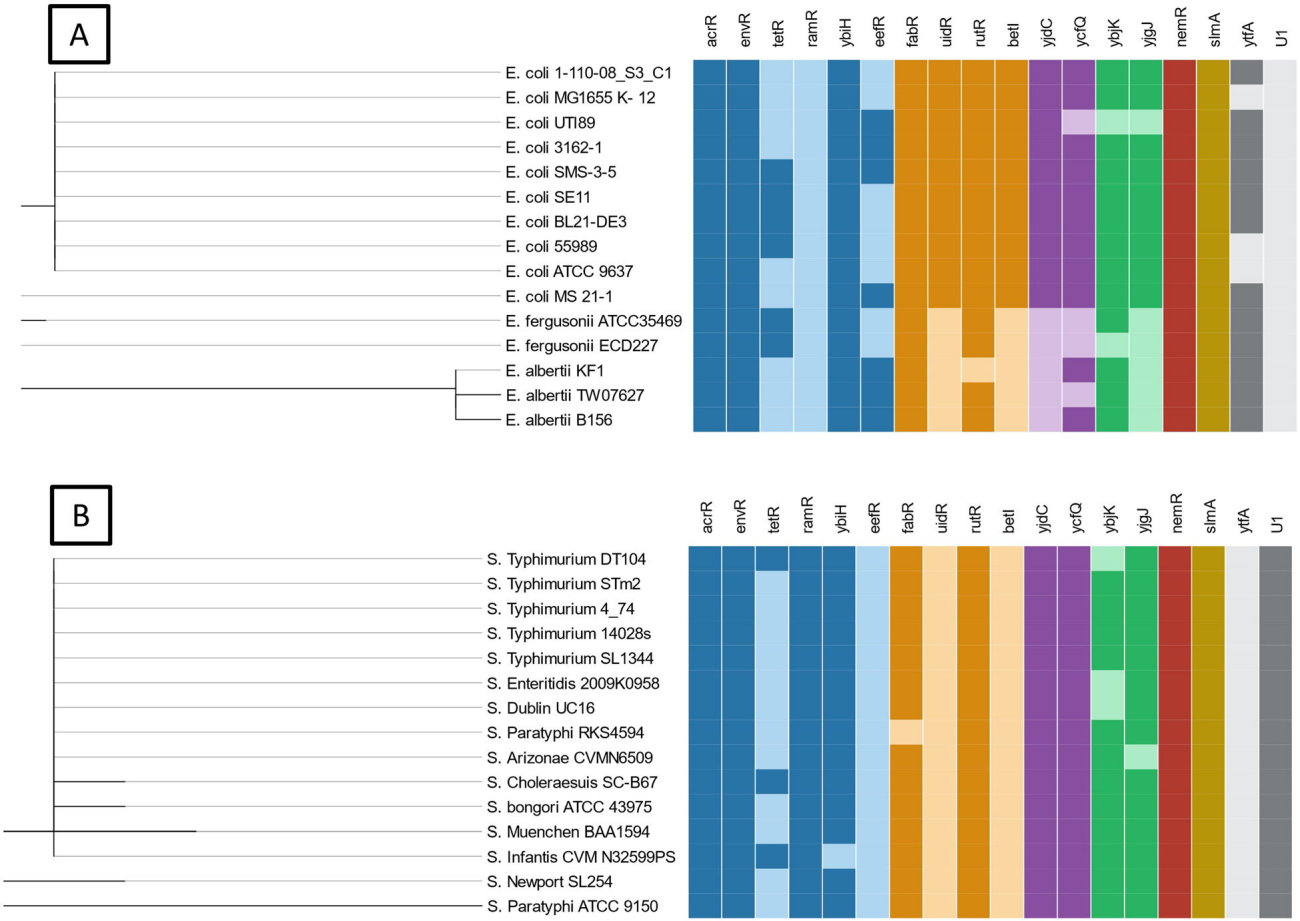
Patterns of TFTR presence/absence across *Escherichia* and *Salmonella* strains. TFTR presence/absence across strains of *Escherichia* (**a**) and *Salmonella* (**b**). Colours of squares indicate proposed function of TFTR, with darker colours indicating presence of the gene in the given strain and lighter colours indicating the gene is absent

A median number of 14.5 TFTRs were identified in *E. coli*. Sequences of *nemR*, *slmA, ybiH*, *envR*, *acrR*, *uidR*, *rutR*, *fabR*, *betI* and *yjdC* were present in all strains of *E. coli*. A further six (*ytfA*, *tetR*, *eefR*, *ycfQ*, *ybjK* and *yjgJ*) were present in some, but not all strains of *E. coli* (Fig. 2). Strain SMS-3-5 contained the highest number of TFTRs (*n* = 16) and strain UTI89 the fewest (*n* = 12). A further two species within the *Escherichia* genera (three strains of *E. albertii* and two strains of *E. fergusonii*, Table 2, see methods) were searched for TFTR genes. These strains contained significantly fewer TFTRs than the *E. coli* strains (Student’s t test *p* < 0.001), with *E. coli* strains having an average TFTR number of 14 versus 10 for the *E. albertii* and *E. fergusonii* strains.

**Table 2:** *Salmonella* and *Escherichia* strains in this study. The nomenclature (genus, species, serovar and strain), accession and number of TFTR sequences are listed for all strains of *Salmonella* and *Escherichia* in this study

Genus	Species/ species and serovar	Strain	NCBI Tax ID	Number of IPR001647 hits
*Salmonella*	*enterica* serovar Typhimurium	DT104	85,569	13
*Salmonella*	*enterica* serovar Typhimurium	STm2	1,218,144	13
*Salmonella*	*enterica* serovar Typhimurium	4_74	909,946	13
*Salmonella*	*enterica* serovar Typhimurium	14,028 s	588,858	13
*Salmonella*	*enterica* serovar Typhimurium	SL1344	216,597	13
*Salmonella*	*enterica* serovar Enteritidis	2009 K0958	1,192,586	12
*Salmonella*	*enterica* serovar Dublin	UC16	1,192,688	12
*Salmonella*	*enterica* serovar Paratyphi	RKS4594	476,213	12
*Salmonella*	*enterica* serovar Arizonae	CVMN6509	1,395,108	12
*Salmonella*	*enterica* serovar Choleraesuis	SC-B67	321,314	14
*Salmonella*	*bongori*	ATCC 43975	54,736	13
*Salmonella*	*enterica* serovar Muenchen	BAA1594	1,079,477	13
*Salmonella*	*enterica* serovar Infantis	CVM N32599PS	1,439,843	13
*Salmonella*	*enterica* serovar Newport	SL254	423,368	13
*Salmonella*	*enterica* serovar Paratyphi	ATCC 9150	295,319	13
*Escherichia*	*coli*	55,989	585,055	14
*Escherichia*	*coli*	ATCC 9637	566,546	13
*Escherichia*	*coli*	BL21-DE3	469,008	15
*Escherichia*	*coli*	MS 21–1	749,527	15
*Escherichia*	*coli*	SE11	409,438	15
*Escherichia*	*coli*	SMS-3-5	439,855	16
*Escherichia*	*coli*	3162–1	1,281,200	15
*Escherichia*	*coli*	UTI89	364,106	12
*Escherichia*	*coli*	1–110-08_S3_C1	1,444,132	14
*Escherichia*	*coli*	MG1655 K-12	511,145	13
*Escherichia*	*albertii*	TW07627	502,347	10
*Escherichia*	*albertii*	B156	550,693	11
*Escherichia*	*albertii*	KF1	1,440,052	10
*Escherichia*	*fergusonii*	ATCC35469	585,054	10
*Escherichia*	*fergusonii*	ECD227	981,367	9

Six TFTRs (*nemR*, *slmA*, *ybiH*, *envR*, *acrR* and *fabR*) were present in all tested strains of the *Escherichia* genus. Of these regulators, the majority are involved in the removal of toxic compounds through either regulating efflux (AcrR, EnvR and YbiH) or, in the case of NemR, activating enzymatic pathways. The TFTRs *uidR, betI* and *yjdC* were present in all *E. coli* strains, but were not present in all *Escherichia* strains searched. In contrast, these same three TFTRs were absent in all strains of *E. fergusonii* and *E. albertii.* In addition to these, all *E. fergusonii* strains also lacked *eefR, ycfQ* and *yjgJ* and *E. albertii* strains lacked *tetR*. All strains of *E. fergusonii* and *E. albertii* have the *ytfA* gene in all strains. In addition to these, *E. albertii* also have *ybjK* and *eefR* and all strains of *E. fergusonii* have *tetR.* Both nodes containing *E. fergusonii* and *E. albertii* also contained fewer TFTRs per strain compared to *E. coli*.

### TFTRs of *S.* typhimurium and *Salmonella* species and serovars

All strains of *S.* Typhimurium had 13 TFTRs and all but one strain, DT104, had the same TFTRs present (Fig. 2). The *tetR* gene was present in DT104 but *ybjK* was absent.

A further 9 strains of *S. enterica* of 7 different serotypes (Arizonae, Dublin, Enteritidis, Choleraesuis, Infantis, Newport, Paratyphi) and one strain of species *S. bongori* were searched for TFTRs. As with *S.* Typhimurium, the range of TFTRs in the *Salmonella* genus did not vary considerably (*n* = 12–14), with *S.* Choleraesuis strain SSC-B67 having the most TFTRs (*n* = 14). Nine TFTRs *acrR*, *envR*, *nemR*, *slmA*, *ramR*, *rutR*, *ycfQ*, *yjdC* and *U1* were present in all strains of the *Salmonella* genus. As in *Escherichia*, the most frequent biological role of these core TFTRs is efflux regulation, with 3 core TFTRs of *Salmonella* (AcrR, EnvR and RamR) being involved in the regulation of multidrug efflux systems. Two TFTR genes were identified (*ramR* and *U1*) which were not present in any *Escherichia spp.* strain in this study. All nodes of the *Salmonella* tree contained the same TFTRs apart from *S. arizonae* which lacked *yjgJ*. This is unsurprising as most *Salmonella* strains included here are serovars within the *S. enterica* species and do not show large variation in either the number or type of TFTRs.

### TFTR number increases with genome size (Mb)

**Fig. 3:**
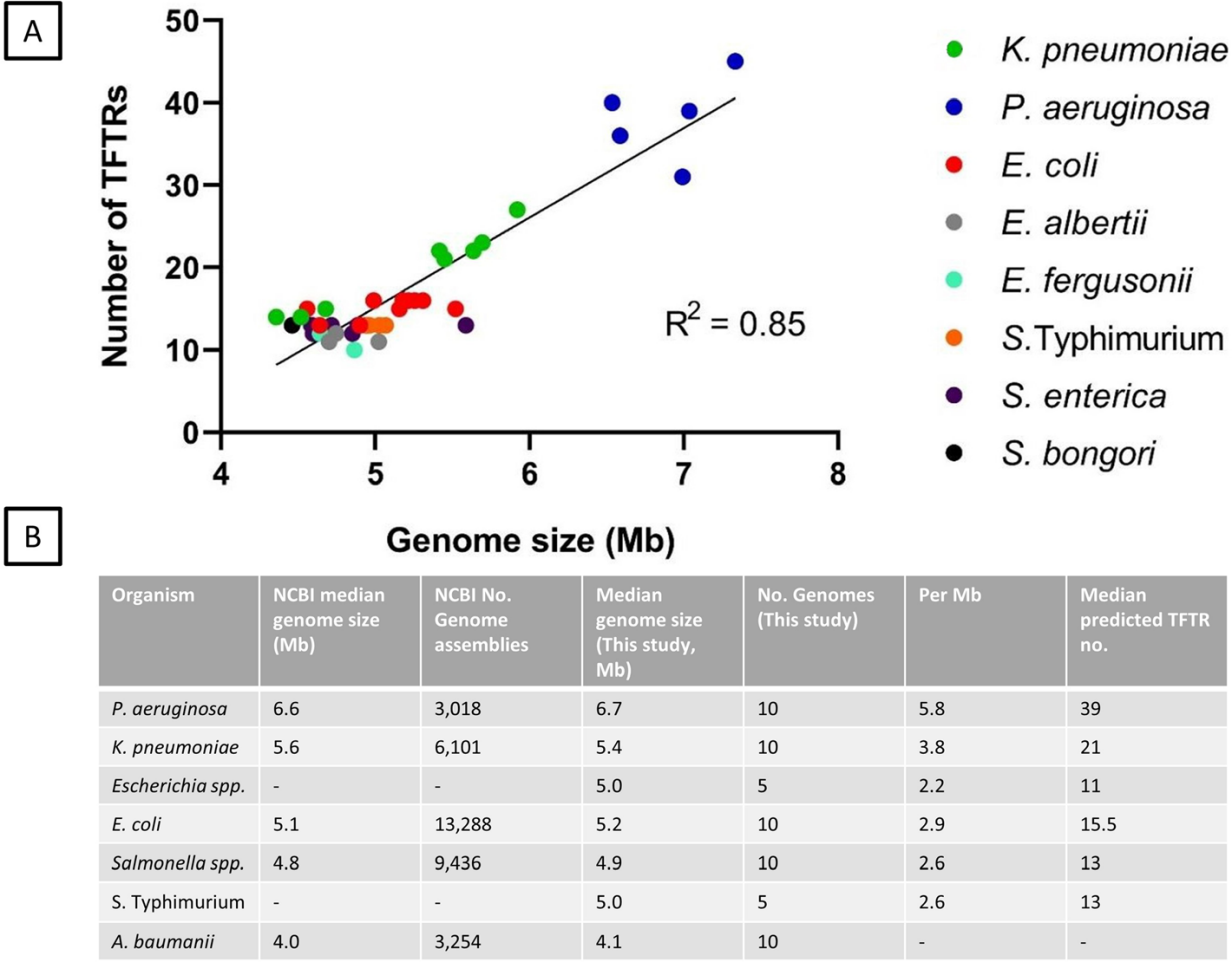
Genome size is positively correlated with the number of TFTRs. **a** TFTR number varied between strains, species and genera of bacteria but was significantly positively correlated with genome size (Mb). The largest range of TFTR number was seen in *Pseudomonas spp.* and the smallest in *S.* Typhimurium. **b** Table describes median genome sizes and n = TFTRs in this study versus NCBI database. The median genome sizes were compared to genomes in this study to check that the genomes selected had a median genome size which is representative of the wider population of isolates. The number of predicted TFTRs was calculated by searching Interpro for IPR001647-containing sequences as previously described. A full list of strains used to produce this figure are available in Additional file 1 and data used to create this figure can be found in Additional file 3

The number of bacterial regulators is known to increase with genome size [1] and TFTR number is known to be positively correlated with genome size in *Mycobacteria* [5]. Here, we show that TFTR number is significantly positively correlated with genome size for a range of bacterial species (R^2^ = 0.85, *p* < 0.01) (Fig. 3). The median genome sizes and TFTR numbers in this study were also comparable to the large number of genomes deposited on the NCBI database (Fig. 3b), validating the methodology used here. *P. aeruginosa* has both the largest median genome size and predicted TFTR number (median = 39, range 36–45). All *S.* Typhimurium strains had 13 TFTRs whereas the *Salmonella* genera had a small range of 12–14 TFTRs. *E. coli* strains had a slightly larger range of 12–16 TFTRs than *Salmonella* and the *Escherichia* genus as a whole had a range of 9–16 TFTRs. There was a significant difference in the number of TFTRs found in *E. albertii* and *E. fergusonii* versus *E. coli* and *Pseudomonas spp.* versus *P. aeruginosa,* with the *E. coli* and *P. aeruginosa* strains having a higher number of TFTRs. It is not known whether the number of targets of TFTRs also increases in larger genomes. As many TFTRs have multiple targets this is difficult to ascertain, and it is also possible that targets for individual TFTRs vary between bacterial species.

### Biological roles of TFTRs of *Escherichia* and *Salmonella*

**Fig. 4:**
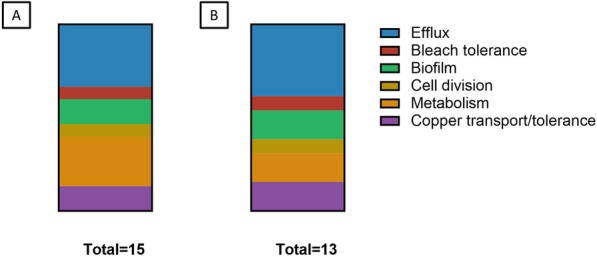
Biological roles of TFTRs in *Escherichia* and *Salmonella.* Proportion of TFTRs predicted to regulate various processes in (**a**) *Escherichia spp.* and (**b**) *Salmonella spp*. Based on the function assigned from literature search (Table 1). *Escherichia spp.* have two additional TFTRs involved in regulating metabolism. No other differences between TFTR function in *Escherichia* and *Salmonella* were observed

There were five TFTR genes found in all *Salmonella* and *Escherichia* searched here: [1] Bleach response regulator *nemR*, Efflux regulators [2] *acrR*, [3] *envR* and [4] *ybiH* and nucleoid occlusion factor [5] *slmA*. In order to classify the TFTRs by role, existing literature was searched for evidence of the regulatory targets and ligands of all TFTRs identified in *Escherichia* and *Salmonella*. Efflux regulation was the most frequent TFTR function (*n* = 6) and the majority of TFTRs which are core in both *Salmonella* and *Escherichia* are efflux regulators. *Escherichia spp.* had two extra TFTRs which regulate metabolism, but there were no other differences in the distribution of TFTR role between these genera (Fig. 4).

Data on the function of TFTRs was then combined with data on the presence/absence of these genes throughout the *Escherichia* and *Salmonella* genera (Table 1). In addition to the five genes conserved in all Gram-negatives tested here (*acrR*, *envR*, *nemR*, *slmA* and *ybiH*), two were core to *Escherichia* (*fabR* and *rutR*) and a further four (*ramR*, *U1*, *ycfQ* and *yjdC*) were core for *Salmonella*. Nine TFTRs are, based on current available literature, single-target regulators. A further seven TFTRs have been shown to either bind upstream of, or affect the transcription of, multiple genes. RutR and YbjK are known activators of at least one of their target genes [11, 35]. Nucleoid occlusion factor SlmA has no known transcriptional regulatory activity in *E. coli* but is a known activator in *V. chloerae* [25].

Certain TFTRs are genera-specific, e.g. the *eefR* gene was not present in any *Salmonella* strains and *ramR* is absent in *Escherichia* strains. TFTRs conserved throughout a genera are denoted as ‘core’ and all other TFTRs are therefore ‘accessory’ for this same genera. Therefore *Salmonella* and *Escherichia* have their own set of core and accessory TFTRs. The percentage carriage of each accessory TFTR was calculated for strains of both genera. Strains lacking the *eefR* gene were also found to lack *eefA* and *eefB,* components of the EefABC efflux system in *Enterobacter* (Additional file 1).

We were unable to collect information on two regulators (YftA and U1) and the sequences of the unidentifiable TFTR are in Additional file 2.

### Sequence variation is related to predicted biological function

The biological roles of many TFTRs in this study are known in *E. coli*, but it is not known if the targets, ligands or functions of TFTRs are genera, species or even strain-specific.

TFTRs which regulate efflux, bleach survival and biofilm formation and dispersal have significantly higher percentage variance (Student’s t test *p* = 0.01) than those involved in regulating cell division, metabolism or copper transport. There was no significant difference in level of TFTR variation between *Escherichia* and *Salmonella*. The lowest variance was seen in nucleoid occlusion factor SlmA.

### Sequence variation is gene and organism- dependant

**Fig. 5:**
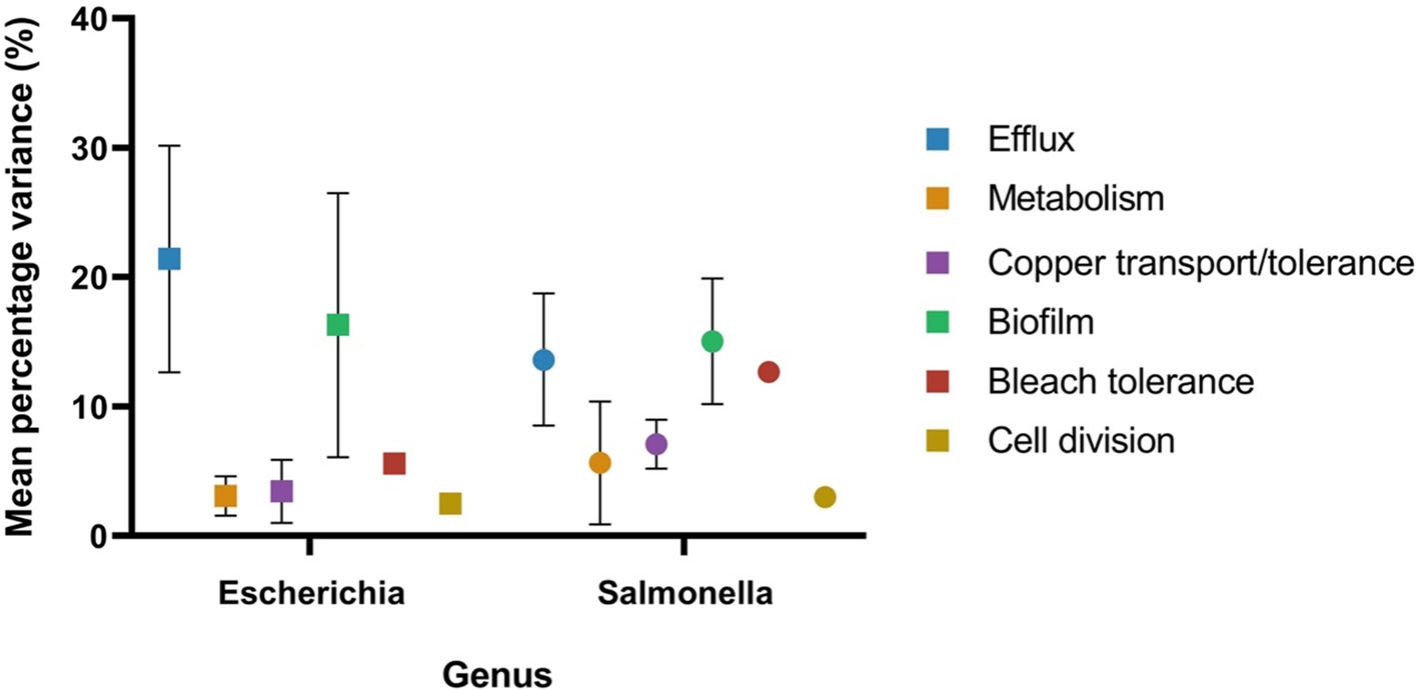
Mean percentage variation in TFTRs grouped by biological function. Percentage sequence variation of TFTRs grouped by function with standard error of the mean. TFTRs regulating efflux regulation, bleach survival or biofilm formation/dispersal have significantly higher percentage variance (Student’s t test *p* = 0.01) than those involved in cell division, metabolism or copper transport/tolerance. This was not a genera-dependant effect, with no significant difference between percentage variance of TFTRs between *Escherichia* and *Salmonella* genera

**Fig. 6:**
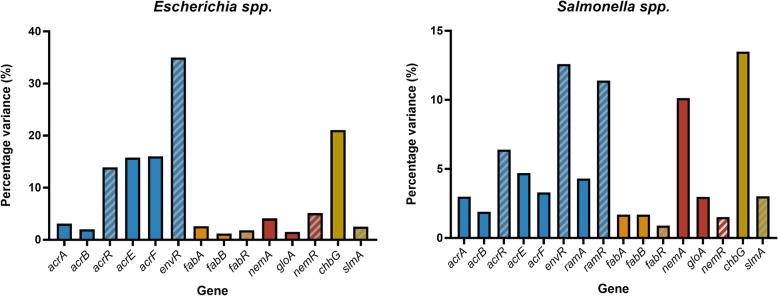
Percentage sequence variation in TFTRs and their targets. Percentage variation in amino acid sequence in TFTRs and their target genes in *Salmonella* and *Escherichia* generated from the sum of polymorphisms after alignment of sequences of the listed genes for each genera

As the sequence variation of TFTRs was shown to vary due to function (Fig. 5), the percentage variation in the TFTR target genes was also investigated and compared to variability of the regulator, in order to ascertain if this could be a function or regulator-specific effect. The percentage variation in TFTRs is shown below (Fig. 6).

There was no clear pattern in how level of variation in the regulator sequence relates to variation in target gene sequence. Sequences of *acrR* were more varied than the operon it regulates, *acrAB*, whereas *fabR* was less variable than *fabAB.* The amount of variation seen in a regulator and its target(s) also varied between genera. For example, there was higher variation in the *acrEF-*envR sequences in *Escherichia.* However, for most other regulator/target pairs, such as *fabR-fabAB,* there were no differences between the genera. Some gene homologs may therefore be under similar levels of selective pressure resulting in comparable levels of variance in different genera.

## Discussion

The number of genes encoding transcription factors varies between bacterial species and this variation depends on both genome size and bacterial lifestyle, with small-genome, niche-restricted species having fewer transcriptional regulators [43, 44]. Conversely, bacteria with large genomes and varied lifestyles such as *Pseudomonas* species contain the largest number of regulatory genes of bacterial genomes studied to date [45]. Data here supports the observation by others that TFTR number positively correlates with genome size [5] and shows that this trend exists throughout *Escherichia* and *Salmonella* in addition to other Gram-negative species.

The inclusion of pathogenic, environmental and laboratory strains, makes the results reported here more representative of the genera as a whole. Strains and species of *Salmonella* and *Escherichia* show variation in the number of TFTRs present, thus even the most recent of evolutionary events are selecting for or against the conservation of certain TFTR genes.

*Salmonella* species tested here were (aside from one strain) serovars of species *S. enterica* and therefore it is expected that these strains did not show significantly different TFTR numbers. However, the *E. coli* strains had significantly more TFTRs than the other species in the genus, *E. fergusonii* and *E. albertii.* All three of these *Escherichia* species have broad host ranges as they are able to colonise and cause infections in both humans and animals [46, 47]. Both *E. fergusonii* and *E. albertii* are emerging enteropathogens [48] [49]. It is possible that the differences in regulatory genes reflect the different lifestyles and virulence of these species.

TFTRs are frequently thought of as simple, single-target negative regulators, however, some have been shown to have multiple targets (e.g. EnvR) [9]. Some TFTRs can be both activators and/or repressors (e.g. MtrR and MerR) or can repress or activate multiple targets (e.g. *glnE* [6] [50]). Recent work by Shimada et al.*,* demonstrates that, for multiple classes of transcription factors, single-target function may be the exception, not the rule [51].

Of the three TFTRs found to be core across the Gram-negative species studied (AcrR, EnvR and NemR), two are regulators of efflux (AcrR and EnvR) and the other promotes bleach tolerance (NemR). This was surprising as it was expected that TFTRs which regulated processes with implications for virulence would likely vary more throughout Gram-negative bacteria. It has been reported that up to 25% of known TFTRs act as regulators of efflux systems [4]. Consistent with this, 33% of TFTRs were predicted to regulate efflux systems in this study. However, when only considering TFTRs found in all strains the majority were efflux regulators. Thus, the most widespread TFTRs in Gram-negative bacteria are those involved in efflux regulation. Efflux is a key mechanism of antimicrobial resistance and the ability to overexpress efflux systems can confer multi-drug resistance, therefore understanding the TFTR regulators of these efflux pumps is essential to better understanding efflux-mediated resistance.

It is possible that the prevalence of TFTRs conserved in this dataset was skewed due to the selection of strains from the WHO priority pathogens list of multidrug resistance species, or perhaps the processes regulated by core TFTRs (i.e. efflux) are more widespread than previously thought. However, the strains used in this study originate from multiple sources (including patient samples, environment and laboratory strains). The number of targets and functions of a TFTR may also either influence the carriage of a particular TFTR gene.

Homologous transcriptional regulators may evolve differentially in different species allowing the acquisition or loss of targets and therefore the further specialisation of the regulator [52]. This means that it is not only the number, but the function, targets and sequence similarity of TFTRs and other regulators which is likely to vary between bacterial species. For example, in one species there may be selective pressure to gain function (i.e. to allow a TFTR to gain an additional target) and it may be expected that some TFTRs have evolved to gain/lose function in accordance with the specificity of the target gene(s) they regulate. The notion that transcriptional regulators can gain targets is not new, for example the CRP regulon of *E. coli* K-12 can be observed to evolve under laboratory conditions in just over 20,000 generations [53].

The plasticity of regulatory pathways is thought to exist partly due to duplication events, through which regulatory genes are duplicated and undergo subsequent specialisation in function [54]. This could also explain some redundancies in regulatory targets and binding sites of TFTRs (for example, AcrR and EnvR both bind to the same site upstream of *acrAB)*. The cross-talk of these efflux systems is not well understood but understanding the conservation of these genes gives insight as to their importance in bacterial species. Moreover, understanding the multiple regulators involved in regulating RND efflux could provide opportunity for drug discovery targets to be identified.

Classifying roles of TFTRs using published literature had unpredicted consequences for this study, including the identification of numerous pseudogenes (Additional file 1) and the identification of the EefABC efflux system in some *Escherichia* species. The TFTR EefR regulates the EefABC RND efflux system in *Enterobacter* species, which is also under regulation by H-NS [28, 29]. This efflux system has not, to our knowledge, been reported in *Escherichia* species. The gene coding for the regulator of the efflux system, *eefR*, was found in four *E. coli* strains and all three *E. albertii* strains, potentially also indicating that the EefABC efflux system is present in these strains of *E. coli* and may therefore be present in other bacterial species.

Higher percentage variation was seen in TFTRs which regulate processes which contribute towards antimicrobial resistance or virulence (i.e biofilm dispersal and efflux) compared to other TFTRs. This may be because variation here can confer favourable phenotypes, which promotes dissemintation and eventually, fixation, of these genotypes. For example, when challenged with antimicrobials, mutations which cause loss-of-function of the TFTR regulator are selected. Polymorphisms in efflux regulators AcrR [14, 15], EnvR [16], RamR [55] and TetR [56], have been reported previously and in this study premature stop codons were observed in the sequences of *envR, acrR*, *acrE* and *acrF* (Additional file 1).

Patterns in sequence variation were not replicated in the target genes of the TFTRs, i.e. the efflux genes tested did not have significantly higher percentage variation than other target genes. This indicates that it is the regulators themselves which are under either positive or negative selective pressure based on the target(s) they regulate and not the particular operon (in the case of locally-acting TFTRs), location with regards to the origin of replication or location within pathogenicity islands. Sequences of *acrR* and *acrAB* showed a similar pattern in both *Escherichia* and *Salmonella* strains, with higher variation in the sequence of the regulator. Similarly, variation in *fabR* and *fabAB* remained low in both genera. Variation was higher in general in *Escherichia* species, although strains within this group were more genetically distant than those tested in the *Salmonella* genera. Some patterns of TFTR and target variation did vary between the genera, notably there was much higher variation in sequences of *envR* and *acrEF* in *Escherichia.* The AcrEF efflux pump shares many substrates with AcrAB and the *acrEF* operon is thought to be H-NS silenced under most conditions [9]. The operon may not be required in many situations, meaning that the whole region encounters spurious polymorphisms and genetic drift.

## Conclusions

The conservation and heterogeneity of TFTRs discussed here highlights the varied and sometimes, underestimated, roles of TFTRs. TFTRs which regulate processes promoting pathogenicity, virulence or multidrug resistance are likely to be more ubiquitous, but contain more sequence variation, throughout Gram-negative bacteria. Our current understanding of TFTRs is largely based on those we have characterised well and for which we have crystal structures, but often leads to the misunderstanding that all TFTRs act in these more simplistic ways.

We propose that the current classification system of TFTRs underestimates the roles of TFTRs and that these proteins often regulate many targets, sometimes using multiple mechanisms.

This is, to our knowledge, the first wide-scale study on TFTRs across Gram-negative pathogens. With rising levels of antimicrobial resistance and limited novel treatment options, we should seek to better understand regulators such as TFTRs which are frequently implicated in multidrug resistant phenotypes.

## Methods

### Identification of TFTR genes in *Escherichia* and *Salmonella*

TFTRs contain a highly conserved helix-turn-helix (HTH) domain at the N-terminus which is denoted as IPR001647 on EMBL-EBI Interpro [57]. Available deposited proteomes of *Salmonella enterica* serovar Typhimurium and *Escherichia coli* (5 strains of *S.* Typhimurium and 10 strains of *E. coli*) were searched for this conserved domain and these protein sequences downloaded. This approach rapidly provides a proxy for how many TFTRs are present due to the high conservation of the HTH domain. Where possible, sequences were annotated with protein name. All proteins had their annotation manually curated using pBLAST [58]**,** producing a database of TFTR protein sequences with confirmed annotations. Orthologues were aligned using Clustal OMEGA [59, 60] to produce neighbour-joining trees of all TFTRs of *S.* Typhimurium and *E. coli*. For example, the sequence of *bm3R1* shared 100% identity with *ramR* and clustered with other *ramR* sequences, but without this alignment these sequences may have been incorrectly assigned an individual identity. This approach also helped to ensure that proteins with multiple names in use (i.e. NemR/YdhM and ComR/YcfQ) were identified as one group and not duplicated.

In order to investigate the variation in TFTR number, type and sequence identity, more proteomes of the wider genera (*Salmonella* and *Escherichia*) were searched in the same way as described above. Table 2 lists all *Salmonella* and *Escherichia* strains included in this study. Any unannotated proteins were searched on pBLAST and all putative TFTRs were aligned with the confirmed ID TFTRs of either *S.* Typhimurium SL1344 or *E. coli* K- 12. TFTRs present in all strains of *Salmonella* or *Escherichia* were denoted as ‘core’ for the given genera. TFTR differentially present in our analysed dataset were denoted as ‘accessory’. Table 1 reviews the known and suspected biological roles of all identified TFTRs and lists whether each TFTR identified was core or accessory for *Salmonella* and *Escherichia*.

### TFTRs in other Gram-negative species

The WHO priority pathogen list comprises the pathogens which most urgently require new antibiotics due to the emergence of multidrug resistant isolates. *Salmonella* and *Escherichia* species are on this list alongside other clinically relevant species such as *P. aeruginosa* and *K. pneumoniae*. The total number of IPR001647 containing-sequences were recorded alongside data on median genome length provided on NCBI. Proteomes, and not genomes, were selected for analyses in this study to enable searching for the specific HTH of TFTRs to prevent false positives. The Gram-negative strains used are listed in Additional file 1.

### Sequence variation of TFTRs and their regulated genes

In order to investigate the variability of TFTRs, all sequences of TFTRs in *Escherichia* and *Salmonella* (*n* = 384) were aligned using Clustal Omega and percentage sequence variation was calculated as the sum of the variable amino acid positions across all sequences of a particular gene in a genera divided by average length of the TFTR gene (Fig. 5). Sequence length was therefore accounted for when considering percentage amino acid variation and TFTRs were grouped based on functions assigned in Table 1.

Known and suspected targets of all the TFTRs identified in *Salmonella* and *Escherichia spp.* were curated by searching available published literature (Table 1)*.* The amino acid sequences of each TFTR were compiled and aligned using Clustal Omega and the number of variable amino acid positions counted. This total was then divided by the mean sequence length for a given TFTR to account for variations in TFTR gene length and multiplied by 100 to give the percentage sequence variance. Here, a variable amino acid position was defined as a position with no consensus amino acid, denoted as either blank, * or ** on Clustal Omega, depending on the possible amino acid substitutions. This process was repeated for the known targets genes of the core TFTRs, excluding targets without conclusive binding studies (i.e. Electrophoretic mobility shift assay) or other proof of binding or regulation (i.e. transcriptomics, ChIP/RNA-seq).

### Phylogenetic analyses

A multiple sequence alignment of the amino acid sequence of AcrB was constructed using MUSCLE [61] for all strains of *Salmonella* and *Escherichia* in this study. The sequence of AcrB varies between strains of *Escherichia* and *Salmonella* and was therefore an ideal candidate for clustering our strains to our desired level of depth. This alignment was then used to construct maximum-likelihood trees with a 100 bootstrap cut-off using MEGA7 [62]. The primary aim of these trees was to separate a small number of very closely-related strains in order to map whether specific TFTRs are present/absent in species of each genera, not precisely map the evolutionary distance between these strains in depth. Phandango was used to combine metadata with the phylogenetic analysis from MEGA7 [18].

## Additional files

Additional file 1: Supplementary material detailing (A) identified pseudogenes, (B) Strains of *K. pneumoniae* and *P. aeruginosa* used in this study and (C) Evidence that strains lacking *eefR* also lack *eefABC. (PDF 673 kb)*

Additional file 2: Supplementary material listing all the unidentified TFTRs from this study. (XLSX 11 kb)

Additional file 3: Supplementary data containing raw data used to produce Figs. 3 and 6. (XLSX 78 kb)

## Data Availability

All data generated or analysed during this study are included in this published article and its supplementary information files.

## Abbreviations

Bp: Base pairs
HTH: Helix-turn-helix
RND: Resistance nodulation division family of efflux pumps
TFTRs: TetR- family transcriptional regulators
