# Supplementary material for: TetR-family transcription factors in Gram-negative bacteria: conservation, variation and implications for efflux-mediated antimicrobial resistance
Source: BMC Genomics. 2019 Oct 12;20:731. doi: 10.1186/s12864-019-6075-5 (PMC6790063; doi:10.1186/s12864-019-6075-5)
Supplement: Supplementary file 1 — Supplementary material detailing (A) identified pseudogenes, (B) Strains of K. pneumoniae and P. aeruginosa used in this study and (C) Evidence that strains lacking eefR also lack eefABC. (PDF 673 kb) [file 12864_2019_6075_MOESM1_ESM.pdf]

Supplementary data

(A) Pseudogenes identified in *Escherichia* and *Salmonella*

The *acrF* gene was not present in *E. coli* 9 (taxid:439855) and *E. albertii* (*taxid:502347).*

A truncated *acrE* gene was present in *S.* Dublin (taxid:1192688) gene accession: EMR51234.1
S. Choleraesuis had both a truncated *acrR* (AAX67232.1) and *acrE* (AAX67233.1). *S.* Typhimurium (*taxid:*85569) had truncated *envR.*

(B) Supplementary table 1: *K. pneumoniae* and *P. aeruginosa* strain used in this study.

Gram negative species	Strain name	NCBI accession	NCBI Genome size (Mb)	Number of IPR001647 hits
K. pneumoniae	342	507522	5.92	27
	30684/NJST258_2	1420013	5.42	22
	IS39	1432561	4.52	14
	IS46	1432553	4.68	15
	ISC21	1432558	4.36	14
	ATCC 700721 / MGH 78578		5.69	23
	ATCC 13884		5.45	21
	909957		5.64	22
	IS43	1269006	4.84	13
P. aeruginosa	PA7	1356855	6.59	36
	DK1	1415629	6.21	n/a
	MTB-1	910265	6.58	n/a
	C-NN2	1262663	6.90	n/a
	VRFPA01	208963	6.99	31
	UCBPP-PA14	1350465	6.54	40
	VRFPA03	1125697	7.04	39
	Stone 130	1402545	7.50	n/a
	BL04	135685	7.33	45
	PAK	1009714	6.41	n/a

Supplementary table 1: The *K. pneumoniae* and *P. aeruginosa* strains used in this study, their average genome sizes (as listed on NCBI) and the number of IPR001647 hits, if available.

(C) Supplementary table 2: Strains lacking *eefR* also lacked *eefAB* efflux genes

**Strain ID of strains lacking *eefR***	Sequence >80% identical or positive to *eefA*	Sequence >80% identical or positive to *eefB*
**585055**	No	No
**566546**	No	No
**469008**	No	No
**409438**	No	No
**1444132**	No	No
**511145**	No	No
**585054**	No	No
**981367**	No	No

Sequences of *eefA* and *eefB* from *E. cloacae* used:

```
>tr|A0A0H3CCT9|A0A0H3CCT9_ENTCC Multidrug efflux periplasmic linker protein EefA OS=Enterobacter cloacae subsp. cloacae (strain ATCC 13047 / DSM 30054 / NBRC 13535 / NCDC 279-56) OX=716541 GN=eefA PE=3 SV=1
```

MMKKITTSIAALLLLTGCDNAQTSAPQRPLPEVGIVTLMSQPVSVVSELTGRTTAAMSAEVRPQVGGIIQKRLFTEGDTVKAGQALYQIDPSSYRAAFDEAAAALKQAQALVQADCQ
KARRYAQLVKDDGVSRQDAEDAKSTCAQDKASVESKKAAQESARINLNWTTVTAPIAGRIGISSVTPGALVTAQQDTALATIRGLDTMYVDLTRSSADLLRLRKQTLASNSDTLSVTLQ
LEDGSTYSEKGRLALTEVAVDESTGSVTLRAVFPNPQHQLLPGMFVRARVDEGIMNDAILAPQQGITRDAKGKATALVVNASNKVEQRQLETGDTYGDKWLVLSGLKAGDRLIVEGT
DKVTAGQQVKAEEMKSSGGNA

```
>tr|A0A0H3CGE3|A0A0H3CGE3_ENTCC Efflux pump membrane transporter OS=Enterobacter cloacae subsp. cloacae (strain ATCC 13047 / DSM 30054 / NBRC 13535 / NCDC 279-56) OX=716541 GN=eefB PE=3 SV=1
```

MFSRFFVRRPVFAWVIAILIMLAGILAIRTLPVAQYPDVAPPSIKISATYTGASAQTLENSVTQVIEQQLTGLDNLLYFTSTSSSDGSVSITVTFEQGTDPDTAQVQVQNKVQQAESRLPT
EVQQSGITVEKSQSNFLLIMGVYDKTDTASSSDIADWLVSNMQDPLARVDGVGSLQVFGAEYAMRIWLDPAKLASYSLMPSDVQSAIEAQNVQVSAGKIGALPSSNAQQLTATVRA
QSRLQTVDQFKNIIVKSQSNGAVVHISDVARVEMGSEDYTSTAKLNGHPAAGMAVMLSPGANALNTATAVKEKIAEFKKSMPEGYDVAYPKDSTEFIKISVEDVIQTLFEAIILVVVV
MYLFLQNIRATLIPALAVPVVLLGTFGVLALFGYSINTLTLFAMVLAIGLLVDDAIVVVENVERIMRDEGLPAREATEKSMGEISGALVAIALVLSAVFLPMAFFGGSTGVIYRQFSVTIISA
MLLSVVVALTLTPALCGSILSHTAPHKKGFFGAFNRFYSKTEHGYQNKVLRALRRSGGMLVIYVLLCGAMGFAMLKLPGSFLPTEDQGEIMVQYTLPAGATSTRTAEVSRQVREWFLT
KEKANTNVIFTIEGFSFSGSGQNAGMAFVSLKNWSERKGDENTAQAIALRATQELSTIRDATIFAMTPPAVDGLGQSNGFTFELMASGGTDRDTLLKLRNQLIGEANQDASLHAVRA
NDLPQMPQLQVDIDNNKAVSLGLSLSDVTDTLSSAWGGTYVNDFIDRGRVKKVYIQGDSDYRAVPSDLNKWYVRGSDSTMTPFSAFATTRWEYGPESLVRYNGSAAYEIQGENAS
GASSGTAMSKMEQLANNLPSGSTWAWSGLSLQEKLASGQAMSLYALSILVVFLCLAALYESWSVPISVILVIPLGVLGAAIAASLRGLNNDVYFQVALLTTIGLSSKNAILIVEFAEAKVA
EGYSLTRAALRAAQTRLRPIIMTSLAFIAGVTPLAIATGAGANSRVAIGTGIIGGTLAATLLAIFFVPLFFVLVKRLFSGKHSNRRS
